# Ethical Dilemmas in Pain Management for Frail and End-of-Life Elderly Patients: Balancing Relief, Autonomy, and Clinical Uncertainty

**DOI:** 10.7759/cureus.96596

**Published:** 2025-11-11

**Authors:** Courage O Idahor, Stephanie I Okeleke, Ogbeide M Aghahowa, Ndidiamaka Ogbonna, Olamide Ogunfuwa, Christabel O Okehie, Uchechukwu T Ozurumba, Gloria C Okparanta, Jennifer A Okonkwo

**Affiliations:** 1 Emergency Medicine, Nottingham University Hospitals NHS Trust, Nottingham, GBR; 2 Public Health, East Tennessee State University, Johnson City, USA; 3 Family Medicine, University of Benin, Benin, NGA; 4 Family Medicine, Edo State University, Uzairue, NGA; 5 Emergency Medicine, Sandwell and West Birmingham NHS Foundation Trust, Birmingham, GBR; 6 Internal Medicine, Uzhhorod National University, Uzhhorod, UKR; 7 Emergency Medicine, Barking, Havering and Redbridge University Hospitals NHS Trust, London, GBR; 8 Surgery, University of Benin, Benin, NGA

**Keywords:** end-of-life care, ethical dilemmas, frail elderly, pain management, palliative care

## Abstract

The management of pain in frail and end-of-life elderly patients represents a significant ethical and clinical challenge in modern healthcare. Demographic transitions toward an aging population and the rising prevalence of frailty and multimorbidity have amplified the complexity of providing safe and compassionate analgesia. Altered pharmacokinetics, polypharmacy, and cognitive impairment often make pain assessment and treatment uncertain, while communication barriers contribute to both underrecognition and undertreatment. Ethical principles of autonomy, beneficence, non-maleficence, and justice frequently intersect in ways that generate tension, particularly when considering opioid prescribing, palliative sedation, or the interpretation of advance directives. Clinicians must navigate these dilemmas within diverse legal and cultural frameworks, where restrictive policies and societal attitudes toward suffering and death strongly influence practice. Interdisciplinary collaboration, involving palliative care teams, geriatricians, nurses, ethicists, and spiritual care providers, enhances shared decision-making and supports patient-centered care, though significant training gaps remain. Recent innovations, including validated observational pain assessment tools, digital monitoring platforms, and artificial intelligence-assisted evaluation, hold promise for improving accuracy and responsiveness in pain management yet raise new ethical concerns regarding depersonalization and equitable access. Addressing these challenges requires clear ethical guidance, supportive policies, and continued research that integrates clinical, legal, and cultural perspectives. This review emphasizes the need for approaches that are compassionate, individualized, and ethically rigorous, ensuring that frail and terminally ill elderly patients receive care that upholds dignity, relieves suffering, and reflects their values at the end of life.

## Introduction and background

Pain management in frail and terminally ill older adults is a central concern in geriatric and palliative medicine because it lies at the intersection of clinical complexity and ethical responsibility. Pain is not merely a symptom but a determinant of quality of life, dignity, and the patient’s sense of self at the end of life. Frailty, which is marked by loss of physiological reserve and heightened vulnerability to stressors, amplifies the risks associated with both untreated pain and pharmacological intervention [1]. The clinical imperative to manage pain in this population is therefore inseparable from moral obligations that extend beyond symptom relief to encompass respect for autonomy, justice in access to care, and the minimization of harm.

Demographic transitions exacerbate this challenge. The global population is aging rapidly, and projections suggest that by 2050, one in six people will be aged 65 years or older [2]. The prevalence of frailty increases with age and is estimated to affect 10% to 15% of community-dwelling older adults, rising to nearly half of those in institutional care [3]. Chronic pain is reported in almost 50% of community-dwelling older adults and is even more common in those residing in nursing homes or receiving hospice care [4]. Among patients approaching the end of life, particularly those with advanced cancer, heart failure, or neurodegenerative disease, the prevalence of moderate to severe pain remains high, often exceeding 70% [5]. These epidemiological realities highlight that the problem of pain in frail elderly patients is both widespread and deeply entrenched, carrying implications for health systems as well as individual clinical practice.

Undertreatment of pain in frail and dying elderly patients remains a persistent problem, shaped by both clinical and ethical factors. Concerns about opioid-related side effects such as respiratory depression, delirium, and constipation often deter clinicians from prescribing effective analgesia, even when the suffering is significant [6]. Fear of regulatory scrutiny, potential diversion, or professional liability adds further hesitation [7]. The result is that many older adults endure avoidable pain in their final months of life, a failure that undermines beneficence and the broader ethical commitment to dignity. Conversely, overtreatment carries its own risks. Excessive sedation, hastened decline, and loss of meaningful interaction with family can result from aggressive pharmacological regimens, raising questions of non-maleficence [8]. The balance between these two extremes is difficult to achieve, particularly in patients with comorbidities that narrow the margin of safety.

The principle of autonomy further complicates the clinical picture. Many older adults express strong preferences about their end-of-life care, with some prioritizing lucidity and meaningful social interaction over complete analgesia. Others prefer maximal pain relief even if it comes at the cost of reduced awareness [9]. Respecting these choices requires clinicians to elicit and honor patient values while balancing them against professional duties and family expectations. The situation is particularly complex in patients with cognitive impairment, where decision-making capacity is compromised and reliance on substituted judgment or best-interest standards is necessary [10]. These dilemmas illustrate how ethical principles can collide, forcing clinicians to navigate competing imperatives rather than apply them in isolation.

Equity in pain management adds another dimension to the ethical debate. Access to palliative care and adequate analgesia remains unevenly distributed both within and across countries. In high-income nations, disparities persist between urban and rural populations, as well as between those with and without access to specialist palliative services [11]. In low- and middle-income countries, barriers are even more profound. Restrictive opioid regulations, inadequate healthcare infrastructure, and limited training of clinicians combine to create what has been termed an “access abyss” in palliative care [12]. These inequities highlight failures of justice, as relief of pain and suffering should not depend on geography or socioeconomic status. Recognition of pain relief as a fundamental human right has gained international traction, but progress remains slow and inconsistent [13].

The interplay of epidemiological trends, clinical challenges, and normative principles therefore shapes the ethical importance of pain management in frail and dying elderly patients. Aging populations ensure that the prevalence of frailty and chronic pain will increase, while the physiological vulnerabilities of these patients make interventions both risky and necessary. The task for clinicians is to reconcile autonomy, beneficence, non-maleficence, and justice in practice, acknowledging that these principles often pull in different directions.

Pain in the frail and dying elderly

Physiological changes associated with aging significantly influence pain perception and analgesic response in frail elderly patients. Alterations in peripheral nociceptor function and central pain modulation contribute to variability in how pain is experienced, with some older adults demonstrating heightened sensitivity and others diminished awareness of painful stimuli [14]. These variations make assessment challenging, as the clinical presentation of pain may not align with objective disease burden. Pharmacokinetic changes further complicate management. Reduced renal clearance and hepatic metabolism slow the elimination of many drugs, increasing the risk of accumulation and toxicity [15]. Changes in body composition, particularly a decrease in lean body mass and an increase in the fat-to-water ratio, alter the distribution of lipophilic and hydrophilic drugs, further modifying their effects [16]. Pharmacodynamic changes, such as increased receptor sensitivity to opioids and benzodiazepines, raise the likelihood of sedation and respiratory depression even at standard doses [17]. These age-related changes underscore why frail and terminally ill patients are uniquely vulnerable to both the undertreatment and overtreatment of pain.

Multimorbidity is common in frail older adults and complicates the evaluation and treatment of pain. Chronic conditions such as osteoarthritis, diabetes, heart failure, and neurodegenerative disease often coexist, creating multiple potential sources of discomfort that overlap in presentation [18]. Distinguishing whether pain originates from musculoskeletal degeneration, neuropathic processes, or visceral pathology is frequently difficult. Polypharmacy, which is highly prevalent in this population, further compounds the problem by increasing the risk of drug-drug interactions and adverse events [19]. For example, the concomitant use of opioids with benzodiazepines or anticholinergics can exacerbate cognitive impairment, falls, and respiratory suppression. Clinicians often hesitate to escalate analgesia for fear of worsening these risks. However, inadequate treatment leaves patients with persistent pain that undermines function and well-being. This balancing act highlights the clinical and ethical challenges of addressing pain in frail and terminally ill elderly patients.

Communication barriers remain one of the most significant obstacles to accurate pain assessment in this group. Cognitive impairment is highly prevalent in frailty and end-of-life care, with dementia and delirium reducing patients’ ability to self-report symptoms reliably [20]. Even among cognitively intact older adults, underreporting of pain is common. Some minimize symptoms due to cultural norms of stoicism, concerns about being a burden, or misconceptions that pain is inevitable with aging [21]. These factors result in systematic under-recognition of pain and its severity. Observational pain assessment tools, such as the Pain Assessment in Advanced Dementia (PAINAD) scale, have been developed to address this gap and rely on indicators such as facial expression, vocalization, and body movements [22]. However, such tools are underutilized in many care settings, leaving pain in non-communicative patients poorly managed. This diagnostic uncertainty complicates ethical decision-making, as clinicians must balance the risk of overtreatment against the equally pressing risk of neglect.

The consequences of inadequately managed pain in frail and dying elderly patients extend beyond physical discomfort. Persistent pain is associated with depression, anxiety, and sleep disturbance, which in turn worsen frailty and reduce resilience to stressors [23]. Functionally, pain impairs mobility and contributes to falls, deconditioning, and dependence on caregivers. Psychosocially, it leads to social withdrawal, loss of engagement, and diminished quality of life. In end-of-life contexts, pain acquires existential significance, undermining dignity and intensifying the suffering of both patients and families [24]. Ethically, the presence of unrelieved pain represents a failure of beneficence and justice, as it deprives individuals of the right to live their final days free of avoidable suffering. Conversely, aggressive pain control can blunt cognition and meaningful interaction, reducing opportunities for closure with loved ones and raising concerns about proportionality and harm [25]. Clinicians face the difficult task of tailoring therapy to relieve suffering while preserving as much function, dignity, and personhood as possible.

## Review

Core ethical principles and their tensions

Respect for autonomy is a fundamental principle in modern medical ethics. However, in the care of frail and dying elderly patients, it is often compromised by cognitive decline, communication barriers, and conflicting interests. Autonomy requires that patients have the capacity to make informed decisions about their treatment and that their preferences are respected and honored. Older adults approaching the end of life frequently face decisions about whether to accept or decline analgesia that may reduce lucidity or hasten decline. Some prioritize alertness to maintain communication with their family, while others value relief from pain, regardless of the sedative effects [7]. In patients with dementia or delirium, decision-making capacity is impaired, and clinicians must rely on advance directives, substituted judgment, or best-interest standards [26]. These scenarios illustrate how respect for autonomy is frequently entangled with paternalistic impulses and family expectations, forcing clinicians to navigate situations in which honoring past or present wishes is fraught with uncertainty.

The principle of beneficence obliges clinicians to relieve suffering, and in pain management, this typically means administering effective analgesia. However, beneficence must be balanced against non-maleficence, the duty to avoid harm. Frail elderly patients are physiologically less tolerant of opioid-related adverse effects such as sedation, delirium, constipation, and respiratory depression [15,17]. The therapeutic window between adequate pain relief and dangerous toxicity is often narrow. Clinicians are therefore forced to consider whether the benefits of aggressive pain relief outweigh the risks of side effects that can diminish quality of life or precipitate clinical deterioration. The doctrine of double effect is sometimes applied in such cases, justifying the use of high-dose opioids or palliative sedation if the primary intention is symptom relief rather than hastening death [27]. While influential in clinical practice, this doctrine is ethically contested, especially when distinguishing intention from outcome is practically impossible.

Justice introduces a systemic perspective, drawing attention to inequities in access to pain relief. In many high-income countries, palliative care services are concentrated in urban centers, leaving rural populations with fewer options for specialist support [11]. In low- and middle-income countries, strict opioid regulations, inadequate training, and resource constraints perpetuate widespread under-treatment of pain [12]. Frail elderly patients, particularly those with cognitive impairment or limited social advocacy, are disproportionately affected by these inequities [28]. The principle of justice requires that all patients have equitable access to pain management; however, the global reality is one of stark disparities in the availability and quality of care. These inequities raise pressing ethical questions about whether healthcare systems and governments are meeting their obligations to protect vulnerable populations from avoidable suffering.

Tensions between autonomy, beneficence, non-maleficence, and justice are rarely resolved straightforwardly. A clinician may respect a patient’s refusal of opioids, thereby honoring autonomy, but risk leaving the patient in severe pain, conflicting with beneficence. Conversely, overriding a patient’s reluctance to accept sedation might align with beneficence but undermine autonomy. Attempts to minimize harm by withholding opioids in frail patients may inadvertently lead to prolonged suffering, conflicting with both beneficence and justice. Structural inequities further complicate these ethical tensions, as clinicians working in under-resourced environments may be unable to provide appropriate pain relief despite recognizing its necessity [29]. The result is a practice environment where ethical principles collide rather than align, demanding case-by-case negotiation rather than formulaic application.

These conflicts demonstrate that principlism provides a useful framework for identifying ethical tensions but is insufficient on its own to resolve them. Ethical practice in pain management for frail and dying elderly patients requires contextual judgment that integrates the principles of autonomy, beneficence, non-maleficence, and justice with sensitivity to clinical realities, patient values, and resource constraints. By understanding how these principles intersect and conflict, clinicians are better equipped to navigate the moral complexity inherent in managing pain at the end of life.

Ethical dilemmas in clinical practice

Opioid prescribing for frail and dying elderly patients remains one of the most visible and contentious ethical dilemmas in clinical practice. Clinicians frequently struggle to balance the legitimate need for effective analgesia with concerns about misuse, dependency, and regulatory oversight. In the context of end-of-life care, fears of addiction are often less relevant than the risk of oversedation, falls, and respiratory compromise [30]. Despite this, opioid stewardship programs and heightened public scrutiny of opioid use have created a clinical environment in which prescribers may hesitate to escalate doses even when pain remains poorly controlled [31]. Families may reinforce this hesitation, expressing fears that opioids will hasten death or render the patient unresponsive, thereby complicating shared decision-making. These concerns, while understandable, often lead to undertreatment of pain and highlight the ethical tension between beneficence, non-maleficence, and autonomy.

Advance directives and informed consent represent another area of ethical difficulty in pain management for elderly patients nearing the end of life. When patients retain decisional capacity, they can clearly articulate their preferences regarding the extent of analgesia or sedation they are willing to accept. In practice, however, cognitive decline, delirium, and fluctuating capacity often undermine the ability to engage in fully informed decision-making [10,26]. In such situations, previously documented advance directives provide guidance, but these documents may be vague, outdated, or absent altogether [32]. Families and clinicians must then rely on substituted judgment or best-interest standards, both of which can generate conflict when patient values are uncertain or interpreted differently by relatives and healthcare providers. The ethical challenge lies in respecting autonomy in a context where autonomy is diminished or inaccessible and where paternalistic interventions may appear necessary to prevent suffering.

Family disagreement is a recurrent ethical dilemma in end-of-life pain management. Conflicts often arise when some relatives advocate for maximal comfort-focused care while others demand escalation of life-sustaining interventions despite the patient’s deteriorating condition. These disputes can stall clinical decision-making and place clinicians in the role of mediators, balancing competing perspectives against what they judge to be the patient’s best interests [33]. Cultural differences further influence family expectations, with some viewing opioid administration as synonymous with giving up, while others interpret the absence of aggressive pain relief as neglect. Clinicians must therefore engage in sensitive communication, often under time pressure, to navigate conflicting values and ensure that the patient’s dignity and comfort are prioritized.

The use of palliative sedation at the end of life introduces some of the most profound ethical challenges in clinical practice. Palliative sedation, defined as the monitored use of medications to intentionally reduce patient consciousness for the relief of intractable suffering, is considered appropriate in cases where symptoms cannot be controlled by other means [34]. The ethical justification often rests on the doctrine of double effect, which holds that the primary intention is symptom relief, even if an unintended consequence may be life-shortening [27]. Nevertheless, critics argue that distinguishing intention from foreseeable outcome is difficult and that palliative sedation risks being conflated with euthanasia in both clinical practice and public perception [35]. Families may struggle with the visible reduction in interaction as sedation deepens, perceiving it as a loss of the patient before death has occurred. Clinicians face the dual responsibility of providing compassionate relief while maintaining ethical integrity in contexts where societal, legal, and cultural boundaries are not always clear.

These ethical dilemmas underscore the inherent complexity of balancing relief of suffering with respect for autonomy, avoidance of harm, and recognition of family and societal values in frail and dying elderly patients. Each clinical encounter involves negotiation between competing priorities, shaped by patient physiology, family dynamics, professional judgment, and external constraints such as law and policy.

Legal and cultural considerations

National and regional laws strongly shape the availability and acceptability of pain management strategies in frail and dying elderly patients. In many high-income countries, frameworks regulating opioid prescribing are designed to limit diversion and misuse but often have unintended consequences for palliative care. Physicians may feel constrained by requirements for detailed documentation, limits on dose escalation, and fear of regulatory scrutiny [36]. In the United States, the Controlled Substances Act and subsequent opioid prescribing guidelines were developed in response to rising opioid misuse but have contributed to reluctance among clinicians to provide adequate analgesia for dying patients [37]. In contrast, some European countries, such as the Netherlands and Belgium, adopt more permissive policies, explicitly integrating palliative care within national health systems and providing greater flexibility in prescribing for end-of-life conditions [38]. These legal variations influence not only the availability of analgesics but also the professional culture surrounding pain management.

In low- and middle-income countries, restrictive laws combined with logistical barriers create profound inequities in access to pain relief. Many nations maintain highly restrictive opioid regulations influenced by international narcotics control frameworks, requiring multiple layers of approval for prescribing morphine even in cases of advanced disease [39]. Concerns over misuse often justify such restrictions but result in widespread undertreatment of pain in dying patients. A report from the International Narcotics Control Board indicated that more than 80% of the global population has little or no access to controlled medicines for pain relief [40]. For frail elderly patients, these restrictions exacerbate suffering and raise pressing ethical questions about whether governments are fulfilling their obligations to protect citizens from avoidable pain.

Cultural attitudes toward pain and death further complicate the legal and ethical landscape. In some societies, enduring pain is interpreted as a sign of strength or moral virtue, leading patients and families to underreport symptoms or resist pharmacological interventions [41]. In others, pain relief is seen as an essential part of dignified dying, with families advocating strongly for aggressive symptom control even at the cost of sedation [42]. These cultural values shape not only patient and family preferences but also professional practices, as clinicians are embedded within the same cultural frameworks. Misalignment between medical recommendations and family beliefs can lead to tension, with clinicians caught between respecting cultural norms and providing evidence-based care.

The emergence of medical aid-in-dying legislation has intensified debate about the relationship between pain management and end-of-life decision-making. Countries such as Canada, Belgium, and the Netherlands permit some form of medical assistance in dying, while others strictly prohibit it [43]. In jurisdictions where aid in dying is legal, the availability of this option may alter the context of palliative care, raising concerns about whether patients are choosing hastened death because of inadequate pain control or loss of dignity [44]. For frail elderly patients, whose suffering is often multifaceted and not solely physical, the line between relief of pain and facilitation of death becomes ethically complex. Critics argue that robust palliative care services should precede the legalization of medical aid in dying, while proponents maintain that both should coexist to maximize autonomy and choice [45].

These legal and cultural considerations demonstrate that pain management for frail and elderly patients nearing the end of life is shaped not only by clinical knowledge and ethical principles but also by broader societal forces.

Interdisciplinary perspectives

Effective pain management for frail and elderly patients nearing the end of life depends on coordinated input from multiple disciplines. Palliative care teams play a central role in this process, integrating physicians, nurses, social workers, and spiritual care providers to address the multidimensional nature of suffering [46]. Their holistic approach ensures that pain is treated not only as a physical symptom but also in relation to psychological distress, social isolation, and spiritual concerns. Geriatricians contribute by evaluating comorbidities, optimizing polypharmacy, and tailoring analgesic regimens to the physiological vulnerabilities of frail older adults [47]. Pain specialists bring expertise in complex pharmacological strategies, nerve blocks, and other interventional modalities, offering options when standard regimens are ineffective. Ethicists help navigate conflicts between patient autonomy, family expectations, and professional duties, while chaplains and spiritual counselors provide meaning-oriented support that can alleviate existential dimensions of pain [48]. This collaborative framework emphasizes the importance of viewing pain management as a shared responsibility, rather than a task delegated to a single discipline.

Communication within and between disciplines is a cornerstone of ethical and effective care. Shared decision-making frameworks encourage open dialogue between clinicians, patients, and families, allowing treatment plans to reflect both medical evidence and patient values [49]. Structured communication tools, such as the SPIKES protocol for delivering difficult news, aid clinicians in discussing prognosis, treatment goals, and analgesic options with sensitivity [50]. Interdisciplinary team meetings provide a venue for discussing complex cases, aligning perspectives, and ensuring that conflicting opinions do not compromise patient care. Failure of communication can lead to fragmented treatment, duplication of efforts, or omission of important considerations such as psychosocial needs or advance care preferences. Interdisciplinary dialogue ensures that decisions are ethically grounded and clinically coherent, even when navigating uncertainty.

Training gaps in ethical reasoning persist as a significant barrier to effective interdisciplinary collaboration. Many frontline clinicians receive limited formal education in ethics. They are left to rely on personal judgment when confronted with dilemmas such as whether to escalate opioid doses or initiate palliative sedation [51]. This variability in ethical competence can lead to inconsistent practices and exacerbate tensions between team members. Interprofessional education programs that emphasize ethical principles, cultural sensitivity, and communication skills can strengthen collective decision-making and reduce moral distress among clinicians [52]. In practice, integrating ethics consultation services into routine palliative and geriatric care provides teams with structured support when facing difficult decisions about proportionality, informed consent, or family disagreement [53].

The role of nurses is particularly critical, as they are often the primary point of contact for patients and families. Nurses continuously assess pain, administer medications, and monitor for side effects, thereby placing them at the intersection of clinical decision-making and patient experience [54]. Their insights into patient preferences and family dynamics often inform broader ethical deliberations within the team. Similarly, social workers play a vital role in addressing financial, logistical, and emotional barriers to effective pain management, helping families navigate the healthcare system and facilitating access to resources [55]. Chaplains contribute by attending to the spiritual dimensions of suffering, which frequently become more pronounced at the end of life. Recognition of these roles ensures that pain management is not narrowly defined by pharmacology but situated within a comprehensive approach to care.

Interdisciplinary perspectives also facilitate the resolution of conflicts that commonly arise in end-of-life care. Disagreements between families and clinicians, or among team members themselves, can stall decision-making and contribute to moral distress. Mechanisms such as family meetings, ethics consultations, and structured conflict-resolution strategies help to mediate disagreements and refocus attention on patient-centered outcomes [56]. These processes are critical when navigating ethically fraught interventions such as continuous palliative sedation or withdrawal of life-sustaining treatment. By incorporating multiple perspectives, teams are better positioned to balance competing ethical principles and to provide care that respects patient dignity while minimizing suffering.

Innovations and future directions

Assessment of pain in frail and cognitively impaired elderly patients has traditionally relied on self-report, but this approach is often unreliable in advanced dementia or delirium. To address this, validated observational tools such as the PAINAD scale and the Doloplus-2 have been refined to capture behavioral indicators, including facial expressions, body movements, and vocalizations [22,57]. These instruments enable clinicians to detect and quantify pain in patients who are unable to verbalize their experiences. Their systematic use improves recognition of suffering and helps guide titration of analgesics; however, implementation remains inconsistent across care settings. Wider integration of such tools into routine geriatric and palliative care represents a practical innovation with significant ethical importance, as it reduces the risk of untreated pain in vulnerable groups.

Digital monitoring technologies are also transforming the landscape of pain management in elderly populations. Wearable devices can track physiological correlates of discomfort, such as changes in heart rate variability, movement patterns, and sleep disruption, which may serve as proxies for pain intensity [58]. Remote monitoring platforms enable continuous assessment outside clinical environments, providing clinicians with longitudinal data that can inform treatment decisions. For frail patients receiving home-based or hospice care, these technologies offer opportunities to detect worsening symptoms earlier and adjust therapy without requiring burdensome hospital visits [59]. The challenge lies in ensuring that such tools are accessible, user-friendly, and validated in older populations with comorbidities.

Artificial intelligence (AI) is increasingly applied to the evaluation of pain and holds promise in contexts where communication barriers complicate assessment. Machine learning algorithms trained on multimodal data, including facial recognition, vocalization analysis, and sensor-based metrics, can identify patterns indicative of pain even in non-verbal individuals [60]. Early studies suggest that AI-assisted systems may outperform human observers in detecting subtle signs of discomfort in patients with dementia [61]. These technologies, however, raise important ethical questions. While they may enhance diagnostic accuracy, there is a risk that overreliance on algorithmic assessments could depersonalize care, thereby reducing clinicians' attentiveness to individual narratives and contextual factors. The ethical integration of AI into geriatric pain management, therefore, requires safeguards that ensure human oversight and preserve the relational aspects of care [62].

The future of pain management for frail and dying elderly patients also depends on the development of ethical guidelines tailored to geriatric contexts. Current frameworks often extrapolate from younger populations or from oncology, neglecting the specific vulnerabilities of older adults with frailty and multimorbidity [8]. Emerging scholarship calls for guidelines that explicitly address the balance between pain relief and preservation of cognitive function, the proportionality of palliative sedation, and the equitable allocation of resources in underfunded settings [63]. These guidelines should integrate insights from palliative medicine, geriatrics, ethics, and public health to provide clinicians with a structured yet flexible basis for decision-making.

Future directions must also address workforce training. The integration of digital monitoring and AI tools will require clinicians to acquire new competencies in interpreting and applying technological outputs in ethically sensitive ways. Interdisciplinary education that combines clinical skills, technological literacy, and ethical reasoning will be crucial in avoiding fragmented adoption or misuse [64]. Moreover, global disparities in access to both technology and opioids necessitate international collaboration to ensure that innovations do not widen inequities but instead extend the reach of effective pain relief to underserved populations. Research into culturally sensitive implementation strategies will be essential, as attitudes toward technology, autonomy, and end-of-life care vary widely across societies [65].

By combining advances in assessment tools, digital monitoring, and AI with the development of ethically informed guidelines, the field is moving toward a more responsive and patient-centered model of pain management.

## Conclusions

Pain management for frail and dying elderly patients is marked by profound ethical dilemmas where the imperative to relieve suffering must be balanced against the risks of harm, loss of autonomy, and inequitable care. Undertreatment erodes dignity, while overtreatment may lead to sedation and diminished quality of remaining life. Clinical complexity, family disagreement, and restrictive legal frameworks further complicate decisions, demanding sensitive and ethically grounded approaches. Innovations in pain assessment and digital monitoring provide valuable tools, but they must be applied with caution to preserve the human dimension of care. The future of practice lies in policies and guidelines that integrate ethical reasoning with clinical evidence, supported by ongoing research and education.

Multidisciplinary collaboration across palliative care, geriatrics, ethics, and psychosocial services is essential to ensure that pain relief at the end of life remains compassionate, personalized, and ethically sound. To advance this goal, healthcare institutions and policymakers should strengthen ethical education, promote evidence-informed decision-making, and allocate adequate resources to support equitable access to palliative care. Establishing clearer legal and professional frameworks can empower clinicians to act confidently within ethical boundaries, ensuring that frail and elderly patients receive care that honors both their comfort and autonomy. Sustained commitment to these principles will help shape a healthcare system that upholds dignity, minimizes suffering, and affirms the value of every life until its natural end.

## References

[1] Clegg A, Young J, Iliffe S, Rikkert MO, Rockwood K (2013). Frailty in elderly people. Lancet.

[2] Department of Economic and Social Affairs (2019). United Nations, Department of Economic and Social Affairs, Population Division. World Population Ageing 2019. World Population Ageing 2019.

[3] Collard RM, Boter H, Schoevers RA, Oude Voshaar RC (2012). Prevalence of frailty in community-dwelling older persons: a systematic review. J Am Geriatr Soc.

[4] Reid MC, Eccleston C, Pillemer K (2015). Management of chronic pain in older adults. BMJ.

[5] van den Beuken-van Everdingen MH, Hochstenbach LM, Joosten EA, Tjan-Heijnen VC, Janssen DJ (2016). Update on prevalence of pain in patients with cancer: systematic review and meta-analysis. J Pain Symptom Manage.

[6] Mercadante S, Arcuri E, Santoni A (2019). Opioid-induced tolerance and hyperalgesia. CNS Drugs.

[7] Berterame S, Erthal J, Thomas J (2016). Use of and barriers to access to opioid analgesics: a worldwide, regional, and national study. Lancet.

[8] Kaasa S, Loge JH, Aapro M (2018). Integration of oncology and palliative care: a lancet oncology commission. Lancet Oncol.

[9] Van den Block L, Deschepper R, Bossuyt N, Drieskens K, Bauwens S, Van Casteren V, Deliens L (2008). Care for patients in the last months of life: the Belgian Sentinel Network monitoring end-of-life care study. Arch Intern Med.

[10] Sessanna L, Jezewski MA (2008). Advance directive decision making among independent community-dwelling older adults: a systematic review of health science literature. J Appl Gerontol.

[11] Clark D, Baur N, Clelland D, Garralda E, López-Fidalgo J, Connor S, Centeno C (2020). Mapping levels of palliative care development in 198 countries: the situation in 2017. J Pain Symptom Manage.

[12] Knaul FM, Farmer PE, Krakauer EL (2018). Alleviating the access abyss in palliative care and pain relief—an imperative of universal health coverage: the Lancet Commission report. Lancet.

[13] Brennan F, Lohman D, Gwyther L (2019). Access to pain management as a human right. Am J Public Health.

[14] Gibson SJ, Lussier D (2012). Prevalence and relevance of pain in older persons. Pain Med.

[15] Mangoni AA, Jackson SH (2004). Age-related changes in pharmacokinetics and pharmacodynamics: basic principles and practical applications. Br J Clin Pharmacol.

[16] Turnheim K (2003). When drug therapy gets old: pharmacokinetics and pharmacodynamics in the elderly. Exp Gerontol.

[17] Bogunovic OJ, Greenfield SF (2004). Practical geriatrics: use of benzodiazepines among elderly patients. Psychiatr Serv.

[18] (2020). Global burden of 369 diseases and injuries in 204 countries and territories, 1990-2019: a systematic analysis for the Global Burden of Disease Study 2019. Lancet.

[19] Maher RL, Hanlon J, Hajjar ER (2014). Clinical consequences of polypharmacy in elderly. Expert Opin Drug Saf.

[20] Achterberg WP, Pieper MJ, van Dalen-Kok AH (2013). Pain management in patients with dementia. Clin Interv Aging.

[21] Schofield P (2007). Pain in older adults: Epidemiology, impact, and barriers to management. Rev Pain.

[22] Warden V, Hurley AC, Volicer L (2003). Development and psychometric evaluation of the Pain Assessment in Advanced Dementia (PAINAD) scale. J Am Med Dir Assoc.

[23] Stubbs B, Binnekade T, Eggermont L, Sepehry AA, Patchay S, Schofield P (2014). Pain and the risk for falls in community-dwelling older adults: systematic review and meta-analysis. Arch Phys Med Rehabil.

[24] Wilson KG, Chochinov HM, McPherson CJ (2007). Suffering with advanced cancer. J Clin Oncol.

[25] Twycross R, Wilcock A, Howard P (2018). Palliative care formulary.

[26] Dworkin RH, Turk DC, Basch E (2011). Considerations for extrapolating evidence of acute and chronic pain analgesic efficacy. Pain.

[27] Sulmasy DP, Pellegrino ED (1999). The rule of double effect: clearing up the double talk. Arch Intern Med.

[28] De Lima L, Pastrana T (2016). Opportunities for palliative care in public health. Annu Rev Public Health.

[29] Cleary J, Powell RA, Munene G (2013). Formulary availability and regulatory barriers to accessibility of opioids for cancer pain in Africa: a report from the Global Opioid Policy Initiative (GOPI). Ann Oncol.

[30] Ersek M, Neradilek MB, Herr K, Jablonski A, Polissar N, Du Pen A (2016). Pain management algorithms for implementing best practices in nursing homes: results of a randomized controlled trial. J Am Med Dir Assoc.

[31] Akdeniz M, Yardımcı B, Kavukcu E (2021). Ethical considerations at the end-of-life care. SAGE Open Med.

[32] Rietjens JAC, Sudore RL, Connolly M (2017). Definition and recommendations for advance care planning: an international consensus supported by the European Association for Palliative Care. Lancet Oncol.

[33] Wendler D, Rid A (2011). Systematic review: the effect on surrogates of making treatment decisions for others. Ann Intern Med.

[34] Cherny NI, Radbruch L (2009). European Association for Palliative Care (EAPC) recommended framework for the use of sedation in palliative care. Palliat Med.

[35] Schildmann E, Schildmann J (2014). Palliative sedation therapy: a systematic literature review and critical appraisal of available guidance on indication and decision making. J Palliat Med.

[36] Anderson KO, Green CR, Payne R (2009). Racial and ethnic disparities in pain: causes and consequences of unequal care. J Pain.

[37] Dowell D, Haegerich TM, Chou R (2016). CDC guideline for prescribing opioids for chronic pain — United States, 2016. JAMA.

[38] Bilsen J, Cohen J, Chambaere K, Pousset G, Onwuteaka-Philipsen BD, Mortier F, Deliens L (2009). Medical end-of-life practices under the euthanasia law in Belgium. N Engl J Med.

[39] Lohman D, Schleifer R, Amon JJ (2010). Access to pain treatment as a human right. BMC Med.

[40] International Narcotics Control Board (2019). International Narcotics Control Board. Narcotic drugs: estimated world requirements for 2019 — statistics for 2017. New York: United Nations. Narcotic drugs: estimated world requirements for 2019 — statistics for 2017.

[41] Blackhall LJ, Murphy ST, Frank G, Michel V, Azen S (1995). Ethnicity and attitudes toward patient autonomy. JAMA.

[42] Kwon JH (2014). Overcoming barriers in cancer pain management. J Clin Oncol.

[43] Emanuel EJ, Onwuteaka-Philipsen BD, Urwin JW, Cohen J (2016). Attitudes and practices of euthanasia and physician-assisted suicide in the United States, Canada, and Europe. JAMA.

[44] Booker R, Bruce A (2020). Palliative sedation and medical assistance in dying: distinctly different or simply semantics?. Nurs Inq.

[45] Quill TE, Back AL, Block SD (2016). Responding to patients requesting physician-assisted death: physician involvement at the very end of life. JAMA.

[46] Meier DE, Beresford L (2008). The palliative care team. J Palliat Med.

[47] Walston J, Buta B, Xue QL (2018). Frailty screening and interventions: considerations for clinical practice. Clin Geriatr Med.

[48] Puchalski C, Ferrell B, Virani R (2009). Improving the quality of spiritual care as a dimension of palliative care: the report of the Consensus Conference. J Palliat Med.

[49] Elwyn G, Frosch D, Thomson R (2012). Shared decision making: a model for clinical practice. J Gen Intern Med.

[50] Baile WF, Buckman R, Lenzi R, Glober G, Beale EA, Kudelka AP (2000). SPIKES-A six-step protocol for delivering bad news: application to the patient with cancer. Oncologist.

[51] Aulisio MP, Arnold RM, Youngner SJ (2000). Health care ethics consultation: nature, goals, and competencies. A position paper from the Society for Health and Human Values-Society for Bioethics Consultation Task Force on Standards for Bioethics Consultation. Ann Intern Med.

[52] Hallin K, Kiessling A, Waldner A, Henriksson P (2009). Active interprofessional education in a patient based setting increases perceived collaborative and professional competence. Med Teach.

[53] Fox E, Myers S, Pearlman RA (2007). Ethics consultation in United States hospitals: a national survey. Am J Bioeth.

[54] Breivik H, Eisenberg E, O'Brien T (2013). The individual and societal burden of chronic pain in Europe: the case for strategic prioritisation and action to improve knowledge and availability of appropriate care. BMC Public Health.

[55] Lloyd-Williams M, Kennedy V, Sixsmith A, Sixsmith J (2007). The end of life: a qualitative study of the perceptions of people over the age of 80 on issues surrounding death and dying. J Pain Symptom Manage.

[56] Schenker Y, White DB, Crowley-Matoka M, Dohan D, Tiver GA, Arnold RM (2024). Resolving disagreements: family conflict in hospice decision-making. J Palliat Med.

[57] Hølen JC, Saltvedt I, Fayers PM, Hjermstad MJ, Loge JH, Kaasa S (2007). Doloplus-2, a valid tool for behavioural pain assessment?. BMC Geriatr.

[58] Majumder S, Mondal T, Deen MJ (2017). Wearable sensors for remote health monitoring. Sensors (Basel).

[59] Hovstadius B, Petersson G, Hellström L, Ericson L (2014). Trends in inappropriate drug therapy prescription in the elderly in Sweden from 2006 to 2013: assessment using national indicators. Drugs Aging.

[60] Fang R, Hosseini E, Zhang R, Fang C, Rafatirad S, Homayoun H (2025). Survey on pain detection using machine learning models: narrative review. JMIR AI.

[61] Hammal Z, Cohn JF (2012). Automatic detection of pain intensity. Proc ACM Int Conf Multimodal Interact.

[62] Topol EJ (2019). High-performance medicine: the convergence of human and artificial intelligence. Nat Med.

[63] Rietjens JA, Korfage IJ, Dunleavy L (2016). Advance care planning--a multi-centre cluster randomised clinical trial: the research protocol of the ACTION study. BMC Cancer.

[64] Wang L, Chen X, Zhang L, Li L, Huang Y, Sun Y, Yuan X (2023). Artificial intelligence in clinical decision support systems for oncology. Int J Med Sci.

[65] Sallnow L, Smith R, Ahmedzai SH (2022). Report of the lancet commission on the value of death: bringing death back into life. Lancet.

